# Subjective Experience of Antidepressant Prescription Among Adolescents With Anorexia Nervosa

**DOI:** 10.3389/fpsyt.2022.770903

**Published:** 2022-04-04

**Authors:** Maude Ludot-Grégoire, Vanille David, Emilie Carretier, Jonathan Lachal, Marie Rose Moro, Corinne Blanchet

**Affiliations:** ^1^APHP, Hôpital Cochin, Maison de Solenn, Paris, France; ^2^Université, PCPP, Boulogne-Billancourt, France; ^3^Université Paris-Saclay, UVSQ, Inserm, CESP, Team DevPsy, Villejuif, France; ^4^Service de Psychiatrie de l'Enfant et de l'Adolescent, CHU de Clermont-Ferrand, Clermont-Ferrand, France; ^5^Université Clermont Auvergne, Clermont-Ferrand, France

**Keywords:** major depressive disorder, anorexia nervosa, antidepressant agent, adolescence, qualitative research

## Abstract

**Introduction:**

Major depressive disorder is considered the most common comorbidity of anorexianervosa in adolescence. Some evidence argues against antidepressant use in this population. Moreover, the fear of being threatened with autonomy and of becoming dependent specific to this adolescent population and at the very core of the anorexic disease, make the proposal of such a treatment difficult to accept. This qualitative research aimed to explore the perspectives of view of adolescents with anorexia nervosa about antidepressants.

**Methods:**

We conducted interviews with adolescents suffering from anorexia nervosa who had been treated with an antidepressant agent during their inpatient or outpatient care at Cochin Hospital. Adolescents were chosen by convenience sampling. Both verbal and written questions were asked to elicit their lived experiences. Interpretative Phenomenological Analysis was used to examine the data.

**Results:**

Fifteen adolescents (13 girls and two boys) were interviewed. We have reached total data saturation. The analysis found two meta-themes, each with two separate themes. (I) Reactions to the proposal of antidepressant with (i) an opposition phase (about the existence of depressive symptoms and about negative preconceptions) and (ii) need to share with family and other teenagers. (II) Reactions to antidepressant use with (iii) ambivalence and an initial perception of coercion, and (iv) effects of antidepressants (psychological effects, effects on the body and on anorexia nervosa and effects perceived through the eyes of others).

**Discussion:**

Despite positive effects, ambivalence toward the treatment remained present throughout the interviews: these adolescents still worry greatly about loss of control and weight gain. Depression as comorbidity seems to be entangled in the denial of disease, cognitive distortion and acquired fearlessness specific to AN. Its existence in adolescence can uncover transgenerational issues, sometimes previously hidden. An orodispersible/drinkable form of antidepressants would facilitate adherence to treatment in this specific population. The intervention of a “patient-expert” could reduce adolescents' anxiety about loss of control. A latency period seems necessary to allow them to understand the prescription at their own level and in the complexity of their anorexic illness.

## Introduction

The DSM-5 (Diagnostic and Statistical Manual of Mental Disorders) defines anorexia nervosa (AN) as a “restriction of energy intake relative to requirements, leading to a significantly low body weight… an intense fear of gaining weight … disturbance in the way in which one's body weight or shape is experienced … or persistent lack of recognition of the seriousness of low body weight” (1). Its prevalence in the adolescent population in France is around 0.5 to 1% (2).

Major depressive disorder (MDD) is significantly associated with chronic somatic diseases in children and adolescents (3). It is the most common psychiatric comorbidity of AN (4–6). Several hypotheses may explain this association: genetic factors these two diseases share (7); alterations of dopamine responsiveness as well as neuroendocrine changes (8, 9); or overdiagnosis of MDD rather than of “depressive moments” during AN (10). Some DSM criteria are common to both MDD and undernutrition, which might also explain this overdiagnosis. For instance, alexithymia is a common symptom in AN that can suggest the possibility of associated or comorbid depression. A study comparing 80 young women with AN and 80 healthy subjects underlined the mediating role of depression on alexithymia in the sick population (11). Nonetheless, depression alone cannot be responsible for all cognitive-affective disturbances in AN. The international literature illustrates the lack of consensus on antidepressant prescription in adolescent populations with anorexia. More precisely, scientific studies report that antidepressant prescription in AN is ineffective (12, 13) or even dangerous (14), while studies of clinical practice show that psychiatric evaluation regularly leads to drug prescriptions (10, 15, 16). A self-reported survey indicates that psychiatrists prescribe psychotropic drugs for 10% of patients with AN (17): olanzapine is prescribed most often, followed by fluoxetine and then sertraline. No randomized controlled trial shows efficacy of any antidepressant in the anorexic population, including after weight restoration (18). In a recent multidisciplinary overview of meta-analyses and systematic reviews, Blanchet et al. (19) concluded there is no impact of antidepressants on weight gain (20–23), and an unclear impact on eating symptoms or psychopathology.

We considered three qualitative studies about psychotropic drugs in adolescence (24–26). Because most of the qualitative studies thus far published about the experience of AN have aimed mainly to explore its related existential upheavals (27), the experience of drug therapies has been examined only sparsely. The first qualitative study described both adolescent and adult experiences of antidepressants (24); it sought to understand why patients find it difficult to continue their treatment. The main themes identified were poor quality interactions with health professionals, insufficient information about antidepressants, and the patients' own experience of side effects. Another qualitative study described how adolescents (with various psychiatric disorders) perceived their psychotropic treatments and observed a discrepancy between the expected effects (depending on perceptions of need for treatment and beliefs about how the medication works) and those observed, which may explain poor treatment adherence (25). Finally, the third study explored the experiences with SSRIs (selective serotonin reuptake inhibitors) of 12 adolescents with depression, a year after their treatment began (26). The four emerging themes were “a threat to autonomy”, “a sign of severity”, “a support, not a solution”, and “an ongoing process of trial and error”. We have found no qualitative studies about the experience of adolescents with AN concerning antidepressants and thus depressive comorbidity. In view of the themes identified in these qualitative studies (with the clear risk of poor treatment adherence) and given our clinical practice of adolescent medicine with AN patients, for whom the question of comorbid depression is regularly raised, we wanted to turn to the adolescents themselves to explore their subjective experiences of antidepressants.

## Materials and Methods

The description of the method follows the COREQ (Consolidated criteria for Reporting Qualitative research) guidelines (see additional material).

### Setting

This single-center exploratory study was conducted in the adolescent medicine department of COCHIN hospital^1^, a multidisciplinary department including an outpatient consultation unit, a day hospital, and a full-time hospitalization unit. The institutional review board (*Comité de Protection des Personnes Nord-Ouest IV* [IDRCB 2019-A02951-56, May 26, 2020]) approved our noninterventional research involving the human person [E3A Experience of adolescents with anorexia nervosa toward antidepressants].

### Sampling and Participants

Two child psychiatrists, ML and VD, conducted this qualitative research with adolescents aged 12 to 25 years, based on the definition of adolescence as the period from the onset of puberty to full independence (28). Although the more used definition of this life stage includes persons aged from 10 to 24 years, cultural and contextual changes can modify it (29, 30). We selected participants according to a convenience sampling, in the outpatient consultation unit or in the inpatient units, as long as they met the inclusion criteria and agreed to participate. We asked patients to participate in this study if they had been diagnosed with restrictive AN by their doctor treating them in the department (according to DSM-5 criteria) and had received SSRI antidepressant treatment during their care, either ongoing or discontinued before this study started.The final inclusion criterion was a body mass index (BMI) > 15. The prescription is usually proposed when patients have reached a suitable nutritional state, for reasons related to safety and pharmacodynamics (31). Particularly, undernourished patients do not have sufficient nutrients to produce serotonin, without which SSRIs cannot be effective. Another explanation could be the deregulation of serotonin receptors in undernutrition. Patients were approached by phone or face to face. The physician gave participants (and, for minors, their parents as well) an information note describing the purpose of the study, the interview procedure, and confidentiality, with a guarantee of anonymization. Participants (and parents of minors) consented to the use of interviews for research, including publication.

### Data Collection

Two researchers (ML or VD) individually conducted a semi-structured interview (see the interview guide elaborated by the research team Table 1) of each participant, lasting 30 to 45 min, after ensuring that the participants (and, if they were minors, their parents) did not object to participating in this research. The interviews were audio recorded and transcribed verbatim. Youths received a written question at the end of the interview, to vary the method of data collection, stimulate the participant's reflective process and enable the adolescents to express their feelings, solicit their memories, and report experiences other than those that came easily to mind while talking.

**Table 1:** Interview guide^*^.

You are/have been receiving care for anorexia nervosa. During your care, your psychiatrist suggested an antidepressant treatment. The purpose of our study is to understand your point of view about the prescription of this treatment. We are going to ask you some questions, feel free to answer them as you wish, there is no right or wrong answer; these interviews will be anonymized.
Oral questions
1. Can you tell me why you are/have been receiving care at the hospital?
2. Can you describe what you thought and how you reacted when your psychiatrist suggested antidepressant treatment?
3. Who did you inform among your family and close friends of this proposed prescription and how did they react?
4. Can you tell us how the first time you took antidepressants went? How did you feel? What were your thoughts and emotions at that time?
5. Can you tell us what the treatment has changed for you, in terms of mood, anorexia, and in the care pathway?
Written question
Can you tell us about an experience related to antidepressant treatment, a moment important for you?

* *Developed in a research team*.

### Analysis

We used a phenomenological interpretative analysis (IPA) (32). This method allows us to access, through discourse analysis, the interconnections between bodily-inscribed experience, emotional reactions, the construction of meanings and finally the oral or written sharing of this experience (32). The researchers (ML and VD) read each interview repeatedly, annotating what was significant in the margins, in terms as close as possible to the wording of the text. Coding units of meaning made it possible for themes to emerge from these codes, that is, for interpretation to begin.

The themes identified were discussed with two other researchers not involved in the interviews (CB and JL). Each interview was analyzed individually. In the next stage, transversal analysis of the interviews enabled meta-themes, that is, themes recurring in multiple interviews, to be identified and new elements to be integrated). The transversal analysis was finally triangulated with another researcher (JL), an expert in qualitative methodology.

## Results

Fifteen patients were interviewed: nine inpatients and six outpatients (day hospital or outpatient clinic), 13 girls and two boys. This enabled data saturation to be achieved and the limited sample allowed us to master the entire corpus and obtain a global vision (33). Table 2 summarizes the participants' weight and some of their therapeutic characteristics. Analysis of the interviews identified two decisive periods in these adolescents' experience: the time when antidepressant treatment was initially proposed and the time when it was actually used. The meta-themes (each with two separate themes) derived from the data.

**Table 2:** Participants' characteristics.

Participants*, **Age (years)**	**Antidepressant agent** **(mg)**	**Inpatient (I)** **or outpatient (O)**	**Weight at the time of interview** **(Kg)**	**Other treatment** **associated** **(mg)**
Maxime, 18	Sertraline 100	I	45.9 BMI 16.66	Aripiprazole 15
Yasmine, 17	Sertraline 75	I	50.3	Lamotrigine 100
			BMI 18.93	(Antiepileptic)
Andréa, 15	Sertraline 100	I	44.2 BMI 17.71	Olanzapine 10
Enzo, 16	Fluoxetine 20	I	58.7 BMI 20.07	Olanzapine 10
Juliette, 17	Sertraline 75	I	47.1	Lamotrigine 50
				(Antiepileptic)
			BMI 19.11	
Jeanne, 22	Fluoxetine 20	O	46.8 BMI 16.77	None
Amélie, 17	Escitalopram 10	O	41.8 BMI 14.98	Olanzapine 15
Raphaëlla, 17	Sertraline 200	I	50.8 BMI 17.58	Olanzapine 10
Manon, 18	Sertraline 100	O	43.8 BMI 15.3	None
Léa, 19	Mirtazapine 15 and Sertraline 100	I	39.4 BMI 17.3	None
Pauline, 18	Sertraline 75: Stopped 14 days before interview	O	42.6 BMI 17.32	None
Mathilde, 16	Citalopram 20	O	54 BMI 19.36	None
Asma, 19	Escitalopram 30	O	36 BMI 16	Olanzapine 5
Lise, 16	Sertraline 125	I	40 BMI 15.51	None
Sophie, 17	Fluoxetine 20	I	44.4 BMI 17.7	Olanzapine 15

* *all names have been anonymized*.

### Reactions to the Proposal for Antidepressant Treatment

The participants had very little to say about the moment that antidepressant treatment was proposed the reasons that led the psychiatrist to suggest it, or the explanations given for it. On the other hand, they did remember their first reactions of opposition and their need to talk about it with both their families and their peers.

#### Opposition Phase

Most adolescents stated that their opposition was based on their disagreement about the presence of depressive symptoms. Nonetheless, when asked for more details about the reasons for this opposition, negative preconceptions about this specific treatment surfaced that might explain it.

##### About the Existence of Depressive Symptoms

These adolescents with AN responded to the suggestion of psychopharmaceutic treatment by minimizing or even denying their depressive affect. Thus, Pauline wondered about such treatment “because an antidepressant is for depression and I wasn't depressed….it was just that…I don't know, everyone can have a little moment of…sadness….” Lise, another teenaged girl, insisted that the term “antidepressant” “doesn't help”. She did not consider herself “depressed” but rather “in low spirits due to my illness”. She concluded “so I don't even know if we can actually call that an ‘antidepressant”'.

For hospitalized teenagers, depressive affect, when identified, is attributed to their medical care, especially when they are hospitalized. As Maxime commented: “it was hard for me to accept being hospitalized again … so I was really down, my mood was really low”. Amélie reported “I was rather sad, I cried a lot…absence, distance of my loved ones, things like that”.

The initial opposition to this treatment was thus expressed very clearly. Lise said, “I told them I didn't want to…” and Amélie stated, “At first I told her NO, I don't want to be on antidepressants, I'm not depressed”. Some adolescents tried to argue, such as Juliette who said she “tried to negotiate so that it wouldn't start”.

##### About Negative Preconceptions

One of the main fears expressed about antidepressants was that they would change the adolescents' personality and make them lose some form of control. Thus, Juliette feared that the treatment would make her “hysterical”, “would change who I am: [my] character…that kind of thing”. She seemed afraid of a mood change and loss of control “[I thought] it would really change me, that I would be happy about everything, that I wouldn't be normal anymore, wouldn't be myself.” Yasmine too expressed the fear that treatment would “change the way I am”.

Behind this, we can perceive the fear of loss of control over anorexia, especially the fear of gaining weight. Léa expressed quite clearly her anxiety about “losing control of the disease a little”. Others reported they often checked out the list of side effects on the package insert. For instance, Yasmine told us that she “still went to see the side effects”, adding, “I was worried about gaining weight”.

Pauline suspected that doctors' aim was for her to gain weight due to the antidepressant treatment: “I thought it was prescribed for that reason too, that I would gain weight.” Finally, Sophie told us that she “went on the internet to look up side effects a little bit” and saw that “in the side effects there was 'getting fatter', so…”

Some of these teens also questioned the antidepressant's mechanism of action. Thus Jeanne found it “a little strange,” adding: “I had trouble understanding how a drug could help me … In fact I didn't believe it, I couldn't see how this thing could change things in my brain.” Other adolescents expressed their overall perspective on treatment. Raphaëlla, for example, said: “I don't like drugs, I try to take them as little as possible.” She then revealed an erroneous belief that confused acetaminophen with antibiotics: “I try to push back the pain every time, I really only take it when I'm in a lot of pain… because it acts on the immune system … and is going to be less effective when I take it because antibodies have created resistance.” Finally, Pauline expressed a fear of a “complete destruction of the brain in the long term” while adding that she was aware of the negative impact of anorexia on the brain: “well, if I go that way, anorexia was destroying me physically and my brain also taking a hit, but then, with the medication, I said Ok, I'm not going to get very far like this.”

Finally, several teenagers expressed fear of addiction to psychotropic treatments. Pauline, for example, asserted: “as soon as you start taking medication, you can't stop like that.” Jeanne said, “I had this fear of becoming an addict”, and Mathilde that “I don't want to be addicted to this”. For Asma, this addictive fear is associated with the fear of being sedated by the treatment, “the fear that it will soften me completely, maybe make me a marshmallow, or an addict.” Just like Amélie, who said she is “afraid that I'll be high”.

#### Need to Share

##### With Family

The adolescents conversed directly with their families after their psychiatrist's proposal. The parents expressed strong concerns, echoing those of the adolescents. Thus, Maxime insisted on his father's reticence: “he doesn't like medication too much, he's against this kind of treatment, which is a little harsh, a little strong, powerful.” Sophie also reported her father's concern about this “difficult, very major treatment, it's not like acetaminophen.” Yasmine, Léa, and Jeanne expressed their parents' fear of “addiction” and “dependence”. For Jeanne, there was even “a kind of rejection in fact” by her parents, “related to a rather serious family history. A bipolar paternal grandfather and another depressed grandfather….” Sophie, too, reported that her grandparents, who had raised her, were very negative due to family history. “Given what had happened in the past, that my father had taken a lot of medications…my grandparents didn't want the same thing to happen again.” She added the impact that these fears had on her: “I think that's what made me resist taking this medication as well”.

In other situations, the parents' discourse was reassuring, echoing positive experiences with their own parents. Thus, Andrea, whose parents were “not at all reluctant,” knew that her maternal grandfather “got depressed when he retired and he had taken an antidepressant…” She added, “he came out of it…so I think it helped him. “Enzo's family”…were all happy and relieved to know that I'm going to get a little bit of help… because my grandmother has been on antidepressants for the last 20 years or so.” He talked to his grandmother who told him that it had helped her a lot and concluded “so I thought, if it helped her a lot, it should help me a lot too”. Finally, Lise summed up her feelings: “Well, my grandmother already had an antidepressant, she says it helped her… she always told me that it could be good, that it could help me… When my mother described her before the antidepressant and I see her as she is now, I tell myself that … it worked well”.

Sometimes, the proposal for antidepressant treatment enabled these teens to learn some previously hidden family history. Enzo, for example, explained that in fact he had not known about his grandmother's: “My father…wanted to keep it from us, but…the doctor asked if there was a psychiatric history…so I learned all this last winter”. Sophie also learned that her mother had suffered from depression “when Dr. K called my mom and said he was going to prescribe this…my mom recognized that it was Prozac and that she had taken it during her depressive episode”.

##### With Other Teenagers

Most of the adolescents interviewed also needed to talk to their peers about antidepressant treatment. Sometimes it was simply to verify that others were receiving this treatment. As Andrea attested: “‘Sometimes we wonder what treatment we have but nothing more.” Other times, they wondered about the benefits and side effects or wanted to confirm their feelings toward the treatment. Thus, Amélie was reassured by a conversation with another girl with anorexia: “She told me ‘I’m also on antidepressants' and that reassured me…she didn't describe any positive effects but she told me ‘I’m on antidepressants' and I saw that she was fine, that she wasn't high”. Enzo also talked with his peers in the hospital and found that “it doesn't sedate us a lot…it's an important drug that not all teens have…it's not insignificant but those who have it are glad they took it.”

### Reactions to Antidepressant Use

Adolescents described the effects they perceived of this treatment once they began taking it.

#### Ambivalence and Initial Perception of Coercion

Several adolescents expressed ambivalence about taking this treatment, especially in a hospital setting that often “made” them take it. Thus, Raphaëlla said that she did not have “too much choice…I accepted but I didn't jump for joy when I was told that I would be prescribed an antidepressant.” She added that “if I could have not taken it, I wouldn't have, but then I took it because it was necessary.” Pauline spoke of “imprisonment” in the hospital: “I couldn't do otherwise, so I took it and then I followed what the doctors told me.”

Sophie expressed her ambivalence by saying that for a long time she “pretended to take the pills” but that ended when she was hospitalized: “necessarily there was a follow-up of what treatment I was taking and then I was obliged to take them…because it was the nurses who gave them to me.” Yasmine and Amélie both described this constraint only at the end of the interview, in writing. Yasmine wrote about “the impression of being stuffed with antidepressants by my psychiatrist,” and Amélie about the feeling of being “trapped, obliged to take the antidepressant.”

The positive effects observed by these same adolescents did not resolve their ambivalence about this treatment, which seemed to persist over time. One teenager summarized it in writing, crossing out a part of it: “Even today, even though I have accepted this treatment and see the effects, I think it changes our personality, our character. In conclusion, the effectiveness of the antidepressant is, in my opinion, largely proven, palpable, and I do not regret taking it for a moment and I thank the doctors for having proposed it to me.” Another teenage girl, Asma, described ambivalent movements, when her dosage increased: “I took the extra 10 mg that day; but the next day, before going to class, I took 20 mg and hid the remaining 10 mg in my hand. A nurse saw me and I went out in the corridor, crying.”

#### Effects of Antidepressants

Five adolescents reported a “placebo effect”, some by name, others indirectly. For instance, for Maxime, “it acts directly, fairly quickly, within minutes, and it fades fairly quickly as well.” Enzo expressed immediate relief, “even though I know that in reality it's not normally supposed to help me on the first day, after 3 weeks I was told…but I already felt it helped from the beginning.” Similarly, Yasmine reported that “I felt better from the first week but they told me it takes at least 4 weeks to really feel the effects.”

Sophie and Raphaëlla, on the other hand, reported a “nocebo effect”. Sophie blamed it on her negative preconceptions: “my grandparents, like me, started with the idea they were not in favor of the antidepressant…I don't know if that accentuated it, the fact of thinking that…necessarily it's bad.” Raphaëlla described it like this: “I don't have the impression that it helps me and therefore the fact of thinking it doesn't help me…that's not going to help me.”

The teenagers all described psychological and physical effects as a result of the prescription; they also reported what those around them perceived and changes noted through the eyes of others.

##### Psychological Effects

The majority of the adolescents we interviewed described positive effects on mood, affect, thought content, and sleep. Juliette noted an anxiolytic effect and the feeling that she had “regained a little bit of self-confidence.” She added that “I sleep much better with the antidepressant.” Manon described “more energy; in fact, it made me want to have projects again…My convictions came back a little bit and it pushed them to come out really well”. Enzo immediately perceived “a decrease in … suicidal ideas.” Yasmine felt “less reserved,” better able to “reach out to others”.

Two adolescents reported mixed and even negative effects. For Raphaëlla, “it didn't work very well either because I'm still a little depressed”. Pauline concluded that “it didn't really change anything. Maybe on relationships with others, but I don't know”. On the other hand, she reported “aggressiveness” because of antidepressants but went on to say “I think I would have been just as aggressive without this treatment.”

Some adolescents described effects on more specific psychological symptoms of AN. For instance, Maxime described its effects on obsessive thoughts: “It keeps me from thinking about the same things all the time. These continuous thoughts, this takes them away and I have fewer intrusive ideas…it's helped me to let go a lot.” Lise talked about a potential effect on dysmorphophobia: “there were more days when I thought I was better, less fat, than days when… well, my self-perception was better than before”. Finally, Juliette wrote that “[t]he antidepressant played a role of enzyme in all of this: it accelerated a reaction that is my recovery.”

##### Effects on the Body and AN

For some of these teenagers, the psychological effects were followed by physical effects. Maxime felt “calmer in my body”, speaking of “psychological and then physical relaxation” that helped him “to not be hyperactive.”

The description of antidepressant effects allowed some adolescents to see the link between mood and AN and therefore between the mood improvement enabled by antidepressants and the improvement of their anorexia. Thus, Amélie reported that after 3 weeks, “it helped me, I was less tired…and then I was smiling more cheerfully…It helped me gain weight…it allowed me to think less about all my anorexic cognitions.” Andrea noticed effects after a month and a half: “My mood was better, I had many more resources to make an effort, and so I could eat better.” For Jeanne, “life was actually simpler. I asked myself a lot fewer questions. I was more able to enjoy moments I spend without my brain…wondering if I'm going to eat, if I'm allowed to, if I've done the right thing.” Finally Lise admitted, “it helped me to see reality and also to lower the little voice in my head telling me 'don't eat this, don't eat that', it was a little less loud.”

Sophie, on the other hand, asserted that “there is no link between the disease and depression.” Accordingly, she argued that “the proof is that when I left the clinic … I was much better in terms of mood and I had managed to stabilize my weight… when I left, I was still under [antidepressant] treatment … and it didn't prevent a relapse of my anorexia.” She concluded that “for me, the two aren't related”.

##### Effects Perceived Through the Eyes of Others

The adolescents often told us that they needed to see through the eyes of others to see treatment effects, both positive and negative. Juliette, for example, said she relied on what the doctors said about her condition “because it's kind of hard to see it, on your own”. She also insisted on the importance of family and friends: “I think you really have to ask questions of those around you, to realize the effect”.

Lise relied on her mother's words, based on photos: “My mother said that even when my dose was not very strong, I was already starting to be 'more alive', I reacted more, I laughed more, with more expressions on my face.”

Pauline, on the other hand, heard the negative effects perceived by her surroundings: “I didn't necessarily feel, but they thought I was more aggressive.”

## Discussion

The experience of these 15 adolescents focused on two different stages: the moment that the psychiatrist suggested antidepressant treatment, and when they began taking these tablets. Most of them initially and emphatically opposed the treatment, before progressively accepting it after discussions with family and friends. At the start of the treatment stage, some adolescents described an initial experience of coercion; they also reported psychological effects, somatic effects, and the importance of the effects perceived by others. Ambivalence toward the treatment remained present throughout the interviews, until the written question, when some expressed persistent doubts more clearly. We propose two lines of discussion: one around the ambivalence about treatment, from the perspective of adolescence and anorexic psychopathology. The second focuses on the ways to care for and improve the therapeutic alliance, emerging from the need for sharing.

### The Ambivalence About Treatment From the Perspective of Adolescence and Anorexic Psychopathology

Adolescence is characterized by progressive empowerment and a willingness to distance oneself from adults; this progression results in ambivalent and sometimes paradoxical movements (34). The help offered is often perceived as threatening the process of empowerment, and the antidepressant treatment that we often offer as a “support” can be perceived as a real threat to the adolescent (35). We can read the initial opposition of adolescents to this treatment in this light, as seen in the qualitative study by Maroun et al. (26) about (non-anorexic) adolescents' perceptions of antidepressants. The first theme to emerge from that study was the perception by adolescents of a “threat to autonomy”. Elements underlying this threat were “a fear of dependence” with antidepressant treatment and “a desire to get out of it on their own (26).”

Opposition to antidepressants takes a particular form in these patients with AN, due to the additional “risk” that the adolescent will “gain weight” or lose control of their weight and body—an essential symptom of anorexia. It is on this subject in particular that there is a confrontation between the adolescent who wants, because of this illness, to keep control of his or her own body and the psychiatrist who, with this prescription, hopes to advance in the cure of anorexia. Sibeoni et al. (35) emphasize the disagreement between adolescents and health professionals about goals of treatment for AN. The goal of health professionals is essentially the disappearance of the physical symptoms of anorexia (weight and behavioral normalization), while adolescents feel that this care focuses too much on somatic aspects and not enough on their psychological distress. The authors insist on the importance of the therapeutic alliance in the treatment of AN in adolescents. This alliance is a key component of adolescent care and depends on both the therapist's clinical expertise and interest in the patient (36, 37).

In addition, the depression itself could be entangled in the denial of disease, cognitive distortion and acquired fearlessness specific to AN. Several interviews clearly demonstrated minimization or even denial of depression, attitudes indicating unfavorability to the proposal for antidepressant treatment. A study conducted in Italy (38) in a sample of 38 adolescents admitted for AN (according to DSM criteria) showed greater denial of anxiety and depressive symptoms (evaluated by the CBCL [Child Behavior Checklist] and YSR [Youth Self Report] scales) among the adolescents with the strongest denial of dietary symptoms.

Consent to treatment and mental capacity to consent to treatment in anorexia are delicate concepts developed and debated in the literature (Turrell plus all coauthors 2011, Elzakkers plus all coauthors 2018). This complicates the question of the relevance of shared decision-making (39) and of the capacity of children and adolescents to make medical decisions for their health (Grootens-Wiegers plus all coauthors 2017).

### Ways to Care for and Improve the Therapeutic Alliance That Emerge From This Need for Sharing

Many of the adolescents interviewed insisted on the importance of verifying, first of all, the condition of other anorexic adolescents taking antidepressants, to ensure it would not change their personality, sedate them, or lead to loss of control of their disease. This observation of others and these conversations appeared more reassuring and more concrete than what their psychiatrist had to say. Use of patient-experts could be useful in this respect and could enable adolescents to take greater ownership of their treatment, improve their alliance with their psychiatrist, and finally improve their adherence to the antidepressant treatment (40, 41). The patient-expert is an individual with a long-term condition whose knowledge and skills are valued and used in partnership with health care professionals (40). The value of patient-experts is recognized in chronic diseases in adults and children (41, 42), and protocols are being developed to assess their interest in the care of AN. A randomized controlled trial of a web-based feedback intervention is currently working on the added value of the patient-expert in the therapeutic education of patients with anorexia (43).

Most adolescents also need to rely on the perceptions of family members, as if to validate their own feelings that they cannot completely trust. As their body perception is disturbed (as in dysmorphophobia), so is their perception of change, which requires special support (44). The family is then central in this mirror effect, especially when the antidepressant treatment refers to depressive episodes in the transgenerational history. This is an opportunity for the adolescent to ask questions about some family events that have been kept quiet and to speak about (during the information and parental consent phases) important family history. Time for this family conversation and this latency period before treatment prescription is important. Some parents remain very ambivalent about the treatment, which of course makes it harder for the adolescent to agree serenely (37).

Our study is the first to specifically explore the experience of prescribing antidepressant treatment to adolescents with AN from their perspective. The adolescents we included had severe AN requiring multidisciplinary care in a specialized center. They were nonetheless interviewed at different stages of their care. Some had other prescriptions: medications (olanzapine) or nondrug treatment (enteral nutrition). The interviews, however, focused on the specifics of antidepressant prescription, and the adolescents were able to easily differentiate between treatments, their indications, and their side effects.

The perceptions of antidepressants in this population of adolescents with AN and significant symptom denial are singular. Although treatment is accepted and positive effects are often observed, ambivalence remains massive. The diagnosis of depression (and the proposal of an antidepressant agent) conflicts with the anorexic position. Adaptations are important for prescriptions in this particular population: an orodispersible or drinkable form would facilitate compliance with treatment.

The adolescents' interviews showed that verification by peers of the innocuity of the treatment is essential and allows them to be less anxious about personality changes. Thus, intervention of a “patient-expert” before the prescription would make the discourse about this treatment more audible; it would be a nonmedical and thus more reassuring intervention. Finally, it is important to be aware of the transgenerational echoes that this depressive issue generates for adolescents. A latency period seems necessary so that the adolescents metabolize these echoes, are reassured when they are too frightening, and understand the prescription at their own level and within the complexity of their anorexic illness. A qualitative research to explore the subjective experience of adolescents suffering from AN about other psychotropic drugs and in particular antipsychotics would be relevant.

## Data Availability Statement

The raw data supporting the conclusions of this article will be made available by the authors, without undue reservation.

## Ethics Statement

The studies involving human participants were reviewed and approved by Comité de Protection des Personnes Nord-Ouest IV [IDRCB 2019-A02951-56, May 26, 2020]. Written informed consent to participate in this study was provided by the participants' legal guardian/next of kin. Written informed consent was obtained from the individual(s), and minor(s)' legal guardian/next of kin, for the publication of any potentially identifiable images or data included in this article.

## Author Contributions

ML-G, VD, JL, and CB contributed to conception and design of the study. ML-G and VD performed the statistical analysis and wrote sections of the manuscript. JL, MRM, and CB supervised the analysis. ML-G wrote the first draft of the manuscript. All authors contributed to manuscript revision, read, and approved the submitted version.

## Conflict of Interest

The authors declare that the research was conducted in the absence of any commercial or financial relationships that could be construed as a potential conflict of interest.

## Publisher's Note

All claims expressed in this article are solely those of the authors and do not necessarily represent those of their affiliated organizations, or those of the publisher, the editors and the reviewers. Any product that may be evaluated in this article, or claim that may be made by its manufacturer, is not guaranteed or endorsed by the publisher.

## Footnotes

^1^APHP, Cochin Hospital, Maison de Solenn, F-75014 Paris, France.
